# Intervention to strengthen caregiving capacities among rural caregivers: a replicable model

**DOI:** 10.3389/fmed.2025.1613937

**Published:** 2025-08-15

**Authors:** Jadith Cristina Lombo-Caicedo, Rosa Margarita Durán-Sabogal, Juan Domingo Palacio-Abello, Francisco Lamus-Lemus

**Affiliations:** ^1^Facultad de Ciencias de la Salud, Universidad del Tolima, Ibagué, Tolima, Colombia; ^2^Facultad de Medicina, Universidad de la Sabana, Bogotá, Cundinamarca, Colombia

**Keywords:** caregiver empowerment, rural healthcare, participatory educational intervention, community engagement, popular health education, distributive education

## Abstract

**Introduction:**

Decades of armed conflict in Colombia have deeply undermined public trust in the health system, particularly within rural regions. The legacy of violence has restricted healthcare delivery in these areas, concentrating services in urban centers and exacerbating geographic and social inequities. Informal caregivers in rural communities, essential yet often overlooked actors in healthcare, face significant challenges due to structural limitations and lack of institutional support.

**Objective:**

This study evaluates a community-based intervention aimed at strengthening the competencies of informal caregivers in rural, post-conflict settings. It further positions caregivers as pivotal contributors to distributed health education models, where care practices must adapt to contextual barriers and local realities.

**Methods:**

Developed using a Participatory Action Research (PAR) approach, the intervention engaged caregivers as co-creators. Drawing from popular education principles, it combined in-person sessions with experiential, culturally relevant materials tailored to rural environments. Ongoing remote support was provided via calls, text messages, and voice notes. The program was evaluated qualitatively through a focus group and 15 semi-structured interviews, analyzed thematically.

**Results:**

Findings revealed four key domains of change: (1) transformation of the caregiving role, (2) personal and technical impact on caregivers, (3) shifts in family dynamics, and (4) recognition of the course as an empowering, community-building experience. Despite barriers such as digital illiteracy, poverty, and low educational attainment, participants demonstrated increased self-efficacy and engagement.

**Conclusion:**

The intervention illustrates the potential of distributed education to reduce rural health inequities. It highlights the power of academic-community partnerships in developing scalable, context-sensitive strategies that strengthen care, foster empowerment, and promote equity in underserved areas.

## Introduction

Colombia’s internal armed conflict, one of the most prolonged in modern history, began in 1944 with bipartisan violence and evolved into a complex confrontation involving insurgent groups (FARC-EP, ELN), paramilitaries, and criminal cartels. Over seven decades of cyclical violence have disproportionately impacted rural areas. Despite the 2016 Peace Agreement with the FARC-EP, structural drivers of conflict—including poverty, inequality, and state absence—remain unresolved. Violence persists, especially in rural regions prioritized for post-conflict recovery (1).

This study was conducted in Icononzo and Chaparral, two municipalities in Tolima Department historically affected by armed groups. Icononzo is home to a Territorial Space for Training and Reincorporation (ETCR), a reintegration initiative for ex-combatants, while Chaparral has been prioritized for the Development Programs with a Territorial Approach (PDET). Both areas continue to experience deep socioeconomic inequities, institutional weakness, and limited access to health and education.

In Colombia, 6.8 million people engage in unpaid caregiving, 85.7% of whom are women. In rural areas, 1.4 million women and 166,000 men assume caregiving responsibilities, with women dedicating an average of 8 h daily versus three for men (2). These caregivers face multiple constraints: geographic isolation, poor transportation infrastructure, and limited institutional support. Just 30% of rural areas have reliable internet and adequate connectivity, limiting access to telehealth services, educational content, and support networks (3–7).

Access to health services in these regions is often inconsistent with rural socio-cultural realities. Caregivers report long wait times, a lack of specialized care, and services that fail to address their needs (4, 8, 9). Financial burdens are also significant, as many caregivers must reduce or abandon paid work, incur in high out-of-pocket expenses for healthcare and transport, and experience economic dependency, all of which contribute to emotional and mental strain (3, 6, 8). Social isolation, lack of institutional support, and multiple caregiving roles—especially among women—lead to chronic stress, burnout, and a sense of institutional marginalization (4, 6, 10).

Enhancing the skills and knowledge of informal caregivers is essential to promote autonomy, improve health outcomes, and build social capital in underserved rural contexts (11, 12). Capacity-building initiatives have demonstrated positive results internationally. In Malawi, a WhatsApp-based program improved psychosocial outcomes among caregivers of people with HIV/AIDS (13). In the United States, cognitive behavioral therapy (CBT) programs for rural caregivers led to improved optimism and reduced anxiety (14). Other multicomponent interventions have shown increased self-efficacy, emotional well-being, and support perception (14, 15). However, some limitations persist, particularly regarding objective caregiving burdens and interpersonal dynamics, underscoring the need for culturally adapted and context-responsive approaches (16, 17).

Multidimensional strategies that integrate psychoeducation with empowerment frameworks appear most effective for enhancing caregiver well-being (18). In Colombia’s post-conflict rural zones, the need for such interventions is urgent and underexplored.

This study aimed to address that gap by implementing a participatory educational intervention with informal caregivers of individuals with chronic illnesses in rural areas affected by armed conflict. The initiative was co-designed with caregivers and led by health sciences faculty, drawing from distributed education principles. By relocating learning to rural territories and emphasizing local relevance, the intervention sought to strengthen caregiving competencies, build academic-community alliances, and contribute to more equitable, sustainable health education models (19).

## Methods

A Participatory Action Research (PAR) approach was used to co-design and implement an educational intervention aimed at strengthening the competencies of informal caregivers in rural, post-conflict regions. Grounded in the principles of popular health education (20), the intervention prioritized caregivers’ lived experiences and was adapted to local rural contexts. Its development involved collaborative input from caregivers, community leaders, and health sciences faculty, ensuring cultural and contextual relevance.

The training, conducted between 2023 and 2024, integrated dialogic pedagogies, experiential learning activities, and territorially adapted materials, combining face-to-face group sessions with remote support via calls, messages, and audio content. To explore caregivers’ perceptions and the meaning they attributed to the experience, a phenomenological qualitative methodology was employed through semi-structured interviews and a focus group.

The study was carried out in two rural municipalities—Icononzo and Chaparral, located in Colombia’s Department of Tolima—both designated as post-conflict areas. Participants were recruited via an open call and snowball sampling, reaching caregivers through local networks. Eligibility criteria included: being an unpaid family caregiver of a person with a chronic non-communicable disease, living in a rural area, having access to a mobile phone, and willingness to participate. Caregivers receiving financial compensation were excluded.

In total, 30 participants enrolled in Icononzo (Territory 1) and 40 in Chaparral (Territory 2). After implementation, 23 participants completed the program in Territory 1 and 25 in Territory 2, with respective dropout rates of 23.3 and 37.5%, largely due to economic pressures, time constraints, and health-related issues.

### Co-creation of the intervention with a popular health education approach

Phase One: As part of the initial phase, home visits were conducted to explore caregivers’ experiences, needs, and expectations regarding the intervention. During these visits, researchers performed semi-structured interviews to assess the knowledge and skills required to care for individuals with chronic illnesses. The interviews were guided by Maslow’s hierarchy of human needs (21), providing a multidimensional framework to identify unmet physiological, emotional, and social needs. Responses were analyzed thematically, revealing recurring concerns and priorities (see Table 1). These findings were later shared with the broader participant group, who collectively validated and refined the topics, shaping the final content of the educational intervention.

**Table 1:** Caregivers’ perceived training needs according to Maslow’s hierarchy of needs.

Dimension	Quotation
Physical	“I want to learn how to give my mom her medicine better.” (2-C-IC-2023)
	“What do I feed her if everything has been taken away?” (1-C-IC-2023)
	“What do I do if my relative falls? Getting them out of the village is very hard!” (3-C-CH-2023)
	“How do I know if a wound is getting worse and when to take my relative to town?” (14-C-CH-2024)
Social	“No one can help me take care of him, I’m scared to leave and something happens.” (2-C-CH-2024)
	“Learning more helps me support my family and others in need.” (4-C-CH-2023)
Emotional	“I’d like to talk about it, because he’s always angry.” (5-C-IC-2023)

Table 1 presents the caregivers’ perceived formative needs, organized into three dimensions—physical, social, and emotional—based on Maslow’s hierarchy. These reflect practical concerns related to caregiving tasks, family support, and emotional strain. Notably, caregivers did not initially identify self-care as a priority, prompting the teaching team to underscore its importance in enhancing caregiving capacities.

Phase two: The second phase focused on co-constructing the intervention content based on thematic priorities identified during the initial needs assessment. Dialogs with caregivers informed the development of each session, fostering an environment where participants could share questions, experiences, and prior knowledge related to caregiving. This participatory design ensured the educational content remained aligned with caregivers’ lived realities and learning interests.

The methodology drew on Paulo Freire’s theory of dialogic education and critical consciousness (22), complemented by the pedagogical expertise of the Health Research Group at the University of Tolima (23–26). Table 2 outlines the thematic areas and strategies employed across sessions.

**Table 2:** Educational content based on popular health education for rural caregivers: shared knowledge, dialogue, and transformative action.

Session	Dialogical construction*	Experiential and reflective learning
1. Recognizing the role of the caregiver	Sharing caregiving experiences	Joint analysis of a case study reflecting rural caregiving reality
2. Understanding illness and complications	Exploring symptoms, myths, and concerns about chronic illnesses	Discussion of clinical cases, identification of warning signs, reflection on personal experiences, and empowerment actions
3. Safe medication administration	Discussing the responsibility of medication use and common difficulties	Reflection and learning about medication administration, empowering other family members
4. Wound care in rural areas	Storytelling of common wound cases; analysis of local practices	Wound care simulations, integrating technical knowledge and traditional rural practices
5. First aid	Sharing rural accident experiences, recognizing emergencies, and the need for an organized response	Emergency simulations using locally available materials
6. The importance of self-care for caregivers	Dialogs on what is appreciated and disliked in the caregiving role; dynamics to identify stress and self-care strategies	Reflection and practice of active breaks and relaxation techniques; recognizing the importance of self-care moments
7. Healthy eating	Discussion of food practices, difficulties, and challenges; explanation of food types and nutritional value	Meal planning with local products; preparation of healthy dishes

*Dialogical construction as defined by Freire, is a horizontal educational process where teacher and learner co-create knowledge through critical dialog, acknowledging themselves as transformative subjects (36).

Each session began with a real-life rural caregiving scenario, followed by experiential learning activities and the use of culturally adapted educational materials. Caregivers were given self-directed exercises and received remote assistance (via calls and messages) to reinforce learning and facilitate real-world application.

Sessions were conducted in public venues within the urban centers of each municipality. To ensure accessibility, transportation stipends were provided. Each session lasted 4 h and included reflective, dialogic, and context-sensitive activities, along with homework tasks designed to stimulate personal reflection and practical implementation.

A range of competency-based strategies were employed to strengthen caregiver capacities. Peer learning was integral to each session, encouraging the exchange of experiences, collective knowledge building, and the formation of a support network. Educational materials were tailored to the realities of rural environments, incorporating both in-person and home-based formats.

Participatory tools were used to explore caregiving challenges and promote collaborative problem-solving. For instance, the “Caregivers’ Network” activity invited participants to reflect on the emotional demands of care and to propose improvements in caregiving and self-care. The “Care Cake” dynamic visually mapped the distribution of care responsibilities within households.

Specific sessions addressed technical skills, such as medication management, where caregivers were trained to develop personalized medication charts with key drug and patient information, enhancing safety and accuracy.

Recognizing literacy barriers, one session used audio messages and photos to teach wound care, allowing participants to narrate their experiences and demonstrate understanding without relying solely on written language.

To monitor engagement and adherence, a systematic attendance and participation log was maintained across the seven in-person sessions. This included tracking session attendance, use of remote support resources, completion of home assignments, and observed application of caregiving practices. Participants were considered to have completed the program if they attended at least 80% of the sessions and demonstrated integration of key caregiving competencies into their daily routines.

Phase Three: In the third phase, a focus group with 16 participants and 15 individual semi-structured interviews were conducted to explore caregivers’ perceptions of the intervention’s impact, relevance, and applicability. This evaluation phase allowed participants to reflect on how the training influenced their caregiving practices and addressed the specific challenges of rural life.

Phase Four: Thematic analysis followed Braun and Clarke’s framework, ensuring methodological rigor throughout the process (27). Data familiarization began with transcription and close reading, followed by inductive coding led by one researcher to identify meaningful units, which were then organized into preliminary themes using analytical matrices and conceptual mapping. A second researcher independently reviewed the coding structure to ensure consistency and clarity. Final themes and subthemes were collaboratively refined, incorporating verbatim participant quotations and contextual interpretations.

The analysis was grounded in a constructivist epistemology, emphasizing the co-construction of meaning through caregivers’ narratives and reflexive researcher interpretation. MAXQDA 2024 software was used to organize and visualize codes and thematic relationships. Additionally, ChatGPT-4.0 (OpenAI) was used to explore the emergence of new categories, though no novel codes were identified. All themes were thoroughly reviewed by the research team to ensure internal coherence and conceptual precision, with any disagreements resolved through discussion and consensus.

### Bioethical considerations

This study adhered to ethical standards for qualitative research and was approved by the Research Ethics Committee of the University of La Sabana (Approval ID: MED-359-2023). It was conducted as part of the broader project, Health Inequities in Rural Colombia: “Characterization of Rural Health Inequalities in Two Municipalities of Tolima as a Basis for a Demonstrative Model Aligned with Community Needs,” funded by Colombia’s Ministry of Science, Technology, and Innovation (Minciencias) and jointly implemented by the University of La Sabana and the University of Tolima.

All participants provided informed consent, were fully briefed on the purpose and scope of the study, and their data were anonymized to ensure confidentiality and uphold their rights throughout the research process.

## Results

Participants who completed the intervention ranged in age from 20 to 70 years, with the majority falling within the 35–64 age group. The cohort was predominantly female (93%). In terms of geographic distribution, 48% of caregivers resided in remote rural areas, while 52% lived in central township zones. Educational backgrounds varied widely, spanning from individuals with no formal education to those with professional training. The care recipients ranged in age from 15 to 94 years.

The qualitative analysis of data from the focus group and post-intervention interviews led to the development of a structured analytical framework comprising categories and subcategories (see Table 3). The main categories represent the overarching thematic axes derived from participants’ narratives, while the subcategories offer a nuanced understanding of caregivers’ lived experiences, the perceived effects of the intervention, and the personal and family-level transformations that resulted from their participation.

**Table 3:** Emergent categories and subcategories from post-intervention analysis.

Categories	Subcategories
Transformation of the caregiver’s role	Learning to care and to self-care
	Empowerment and autonomy
Impact of the course on the caregiver and caregiving	Application of technical knowledge
	Development of skills
	Change in caregiver habits
Change in family and social dynamics	Redistribution of caregiving
	Family unity and communication
	Encouraging care recipient autonomy
	Observable patient improvements
Course value as a training and community space	Relevance of the learning experience
	Peer learning

The full set of themes emerging from the analysis of caregiver narratives is summarized in Table 3, which outlines the interpretive framework constructed through thematic analysis.

The following section details the emergent categories and subcategories, offering a comprehensive view of the complex, multidimensional nature of caregiving for individuals with chronic illnesses in rural contexts.

### Transformation of the caregiver’s role

This category captures the evolving process through which caregivers redefined their identities and caregiving functions as a result of their participation in the educational intervention. The program empowered participants to see themselves as active subjects with rights, emotional needs, and decision-making agency. Through dialogical learning and collective reflection, caregiving was reimagined not merely as a task-oriented obligation, but as a relational and reciprocal practice—one that demands emotional regulation, shared family responsibility, and a foundational sense of self-respect.

#### Learning to care and self-care

This subcategory reflects a key turning point in participants’ conceptualization of their roles. Caregivers began to recognize themselves not only as providers of care, but as individuals equally deserving of attention and support. This shift in self-awareness contributed to the strengthening of caregiving practices by reinforcing the caregiver’s sense of dignity and personal worth. As expressed in participants’ reflections, acknowledging the importance of self-care generated a positive feedback loop of well-being within the patient–caregiver dyad, where the health of one directly influenced the other.

Participant narratives underscored the significance of self-care as a foundational component of effective caregiving. This emergent understanding reflects a transformative shift in the caregiver identity—from one characterized by self-sacrifice to one rooted in mutual care, self-worth, and boundary-setting.

As one participant expressed, *“I’ve learned to value myself, to take care of myself, and understand it’s not just about caring for the patient”* (FOCUS GROUP: 77). Another reinforced the importance of personal limits, stating, *“I’ve learned that I need to care for and value myself, not take on too many duties, and set limits”* (FOCUS GROUP: 87).

This evolving self-perception was further captured in a caregiver’s comment: *“I also learned to value my time. I learned to value myself as a caregiver… I have to be well first in order to provide better care”* (13-C-CH: 4–4). Collectively, these insights emphasize the emergence of a reciprocal care dynamic, wherein the caregiver’s own health and self-recognition are understood as essential to sustaining the health and dignity of the care recipient. The formation of this patient–caregiver dyad, grounded in mutual well-being, marks a significant advance in the conceptualization of caregiving within rural contexts.

#### Empowerment and autonomy

The emergence of personal agency was identified as a key subcategory within the broader theme of transformation. Through the intervention, caregivers began to recognize their right to express needs, request support, and share caregiving responsibilities—actions that marked a significant shift from silent endurance to active participation in their care networks. This process of empowerment extended beyond functional relief; it also held profound emotional significance, fostering a sense of liberation from the isolation, invisibility, and disproportionate burden that often characterize caregiving in rural settings.

One participant reflected, *“I learned I do not have to do it all myself; others can and should help”* (FOCUS GROUP: 44), highlighting a pivotal shift from solitary responsibility to shared care. Another stated, *“I never knew I had the right to speak to my siblings… now I do”* (FOCUS GROUP: 71), emphasizing how the intervention helped reframe family dynamics and open pathways for communication and collaboration. This shift toward autonomy was also reflected in self-perceived well-being: *“I’ve gained weight and feel more at ease… thanks to this course, I let go of the burden I was carrying”* (04-C-CH: 4).

These narratives illustrate the caregiver’s evolving recognition of themselves as subjects of both rights and responsibilities. Learning to express their needs, ask for help, and activate existing support networks was interpreted not as a sign of weakness but as an act of personal reconciliation—with oneself, one’s family, and the person receiving care. This redefinition of autonomy reaffirms the caregiving role as one rooted in dignity, self-knowledge, and shared responsibility—key pillars for sustainable caregiving practices in underserved rural settings.

### Impact of the course on the caregiver and caregiving

This category captures the multifaceted effects of the educational intervention on both caregiving practices and caregivers’ personal development. The impact extended beyond technical knowledge acquisition to include the reconfiguration of daily routines, emotional responses, caregiving attitudes, and self-care behaviors within the rural caregiving context.

#### Application of technical knowledge

Caregivers reported successfully integrating new technical competencies into their caregiving routines. These competencies were perceived as relevant and applicable to the realities of rural life. As one participant noted, *“I’ve learned how to give medication properly—dosage, route, timing”* (13-C-CH: 4). Another shared, *“I have put into practice the care of a venous ulcer—how to clean it, manage it properly, and ensure appropriate nutrition”* (14-C-CH: 4). A third participant reflected, *“I’ve learned how to safely transfer my father and identify bathroom hazards”* (FOCUS GROUP: 30). These examples demonstrate the practical implementation of health knowledge, particularly in settings where professional care is limited. They also reveal how applied knowledge can function as a protective factor for individuals with chronic conditions and help reduce health disparities in rural areas.

#### Development of caregiving skills

Participants described acquiring interpersonal and emotional skills that enhanced their ability to respond to the complex demands of caregiving. For example, one caregiver shared, *“I’ve developed patience and empathy… and learned how to help my relative cope with the illness”* (13-C-CH: 4). Another remarked, *“I discovered hidden skills that are now helping me on this journey”* (15-C-CH: 6). These accounts highlight the adaptive capacity that emerged through experiential learning, emphasizing the relational and emotional dimensions of caregiving.

#### Changes in caregiver habits

The intervention also prompted caregivers to adopt healthier personal habits, recognizing the interdependence between their own well-being and their caregiving effectiveness. As one participant explained, *“I used to forget my own medication; now I’m disciplined… it’s changed my life”* (FOCUS GROUP: 16). Another noted, *“I quit drinking so much coffee and feeling nervous… I have more control now”* (FOCUS GROUP: 129). These behavioral shifts reflect an internalization of the training content and a broader commitment to self-care as an essential caregiving strategy.

Together, these reflections illustrate a process of caregiver empowerment in which newly acquired knowledge is actively applied to enhance both the quality of care provided and the caregivers’ own health and autonomy.

### Change in family and social dynamics

This category captures the relational and collective transformations that occurred within families as a result of caregivers’ participation in the educational intervention. By recognizing the significance of caregiving and developing communication skills, caregivers fostered the redistribution of responsibilities and initiated processes of shared care. These changes reduced the sense of isolation and burden often associated with caregiving in rural settings, while simultaneously strengthening emotional bonds and intrafamilial communication.

#### Redistribution of caregiving responsibilities

This subcategory reflects the caregiver’s increased ability to voice their needs and negotiate caregiving tasks with family members—an outcome closely tied to the empowerment developed through the intervention. As one participant noted, *“Now everyone helps. Before, I was the only one looking after my mom”* (FOCUS GROUP: 71). Another added, *“My sister took over when I had to leave… I learned to delegate”* (FOCUS GROUP: 44). These statements illustrate how the intervention promoted more equitable distribution of labor and encouraged caregivers to seek and accept support, reshaping caregiving as a shared family practice.

#### Family cohesion and communication

While caregiving responsibilities can often lead to tension and emotional strain within families, participants reported improvements in mutual understanding and relational closeness. This shift was facilitated by greater dialog, emotional expression, and appreciation of caregiving as an act of love and solidarity. One caregiver reflected, *“What I love most is caring for my husband… we talk more now, we are closer. I take care of him with very much love and affection”* (06-C-CH: 4). Another observed, *“Now we have lunch together, we share more, and we appreciate each other”* (FOCUS GROUP: 142). These accounts suggest that caregiving, when supported and valued, can catalyze family cohesion and interpersonal growth.

#### Observable patient improvements

Participants also reported notable improvements in the autonomy and emotional well-being of those receiving care, which they attributed to changes in caregiving practices. One caregiver shared, *“She now bathes herself—she did not before”* (FOCUS GROUP: 106–108), while another noted, *“Don Luis now makes his bed, bathes himself, and is happier”* (FOCUS GROUP: 77). These reflections indicate that caregiver empowerment and family engagement had positive ripple effects on the care recipients themselves, fostering greater independence and well-being within the household.

### Value of the course as a training and community space

Participants described the educational intervention not only as a source of practical knowledge but also as a transformative community experience. The course served as a collective learning space where education was closely tied to mutual recognition, shared experiences, and the development of solidarity among caregivers.

#### Relevance of the learning experience

The perceived relevance of the course stemmed from its alignment with real-world caregiving needs. Participants emphasized that the content addressed everyday challenges and provided them with concrete tools to manage chronic conditions, boosting their confidence and reframing their identity as caregivers. As one participant noted, *“This course has helped me… now I know how to respond to hypertension or diabetes”* (05-C-CH: 4). Another reflected, *“In 22 years of caring for my son, we have never had training like this”* (FOCUS GROUP: 135). These statements underscore the course’s value as a long-overdue educational intervention that responded directly to a critical knowledge gap in rural caregiving.

#### Peer learning

Moreover, peer learning emerged as a powerful element of the experience. The opportunity to learn from others in similar situations not only enhanced knowledge acquisition but also fostered emotional support, strengthened community ties, and helped create a safe, trusting environment. One caregiver shared, *“I’m learning a lot from others’ experiences around me”* (02-C-CH: 5), while another highlighted the ripple effect of community solidarity: *“We’ve been supporting each other in* var*ious ways, and for those who have not taken the course but are in similar situations, we have also found ways to help and assist them”* (FOCUS GROUP: 63). These accounts reflect how the course functioned as a space for democratizing knowledge and promoting inclusive, network-based caregiving practices.

## Discussion

Rural caregivers in Colombia face interconnected challenges—including geographic isolation, economic hardship, limited digital literacy, and weak health infrastructure—which compound their caregiving burdens and restrict access to formal support systems (7). These barriers are particularly acute in post-conflict territories, where structural disadvantages accumulate over time. In this context, distributed education emerges as a promising strategy to enhance community care capacity and resilience.

Also known as decentralized or community-based education, this approach grounds clinical and public health training in the lived realities of rural and underserved populations. By embedding learning within local contexts, universities are positioned to address systemic inequities, engage with social and cultural dynamics, and foster a shared sense of responsibility through partnerships with the community.

As emphasized by Gomes and Merhy (20) and Muller et al. (19), academic institutions play a pivotal role in promoting participatory, context-sensitive education within marginalized settings. This study further affirms the university’s role as a driver of territorial equity and social justice. In this framework, distributed education becomes a platform for building synergies among academia, communities, and health systems, particularly in rural areas.

The following discussion is organized around five thematic axes derived from caregivers’ qualitative narratives, which illustrate the intervention’s impact and inform the development of contextually grounded educational strategies in rural health.

### On caregiver role development

The enhanced perception of the caregiver role observed in this study aligns with prior evidence demonstrating the benefits of community-based interventions. One study reported improvements in self-care and coping strategies among rural caregivers, underscoring the importance of addressing the emotional and psychological dimensions of caregiving (28). Similarly, in New Zealand, a lifestyle-focused program effectively promoted healthier habits, illustrating the value of locally grounded approaches to caregiver well-being (29).

In rural Pakistan, a technology-assisted parenting intervention targeting caregivers of children with developmental disorders led to increased confidence and empowerment, significantly enhancing caregivers’ ability to support developmental progress. Comparable to the current intervention, this model demonstrated strong scalability and adaptability in resource-constrained rural settings, reinforcing the potential of distributed education models to strengthen caregiver agency and broaden access to relevant training (30).

### On technical skill development and behavioral change

The observed improvements in technical caregiving skills and health-related behaviors are consistent with findings from previous studies. For instance, a training program for rural African American caregivers of individuals requiring palliative care reported that participants felt better prepared post-intervention, supporting the use of targeted training to mitigate the shortage of specialized healthcare professionals in underserved areas (31).

Given the sociodemographic complexities of rural contexts, community-based educational interventions that enhance practical knowledge and caregiving competencies can be effective strategies for reducing caregiver burden and improving overall quality of life. A study involving 32 rural caregiver-care recipient dyads demonstrated that a culturally tailored, phone-based intervention significantly reduced caregiver burden, showcasing the potential of remote and context-sensitive approaches in regions with limited in-person access (32).

Similarly, an intervention aimed at reducing sugar-sweetened beverage consumption in rural areas was met with high acceptance by caregivers, emphasizing the value of adaptable and behavior-focused strategies (33). Collectively, these studies highlight the effectiveness of flexible, context-aware interventions and reaffirm the critical role of academic institutions in their design, implementation, and sustainability.

### On transforming family dynamics

The intervention led to significant transformations within family systems, particularly through the redistribution of caregiving responsibilities, the strengthening of emotional bonds, and the promotion of autonomy among care recipients. These outcomes reflect the relational impact of empowering caregivers, not only on individual well-being but also on the broader family dynamic.

Comparable findings were reported in a study involving the co-creation of services with rural caregivers, which contributed to emotional support, enhanced self-confidence, and the reinforcement of community networks. That study also identified logistical and infrastructure barriers—such as limited access and connectivity—that mirror the contextual challenges encountered in the present intervention (34).

### On the role of educators and digital health literacy

The intervention, led by university faculty, was positively received by participants and offered valuable insights for the design of future community-based initiatives. The academic involvement and contextually relevant content were highly appreciated, reinforcing the role of universities in co-creating responsive educational strategies.

However, digital literacy emerged as a critical determinant of caregiver engagement and participation. As highlighted in prior research (33, 34), limited proficiency with digital tools can restrict the effectiveness of technology-based interventions in rural areas. These findings underscore the importance of integrating digital literacy training into future virtual or hybrid educational models, ensuring more inclusive and equitable access to learning opportunities.

### Distributed education: a model of impact and sustainability

This experience exemplifies the effective application of distributed education in post-conflict rural settings, demonstrating how academic-community collaboration can enhance both caregiving competencies and the broader social context in which care occurs. It underscores the value of scalable, context-sensitive education models rooted in territorial equity and service-learning principles, fostering knowledge co-construction and sustained engagement between universities and communities (35).

In Colombia, this initiative marks a pioneering effort to bridge academic training with rural realities, advancing human resource development in health grounded in equity, social accountability, and territorial justice.

### Limitations

Several limitations should be acknowledged. Methodologically, the lack of ethnographic observation limited the triangulation of participants’ narratives with actual caregiving practices. Additionally, the absence of member-checking to validate interpretations may have affected the confirmability of the findings.

At the participant level, factors such as low educational attainment, limited digital literacy, and time constraints reduced engagement. Structural barriers—including geographic isolation, transportation difficulties, and poor internet connectivity—further hindered participation in both on-site and remote components.

Finally, institutional limitations within participating universities constrained mobility and logistical support for rural engagement, revealing the need for more adaptable academic structures to effectively implement distributed education models in rural contexts.

## Conclusion

This study demonstrates the effectiveness of community-based educational interventions in enhancing caregiving capacities within rural settings. The caregivers who participated not only developed practical technical competencies but also experienced meaningful personal, familial, and social transformations. These shifts contributed to improved well-being for both caregivers and care recipients, reaffirming the value of integrating education with empowerment in underserved communities.

The findings further underscore the strategic importance of embedding distributed education approaches within the training of the healthcare workforce. By recognizing caregivers as essential actors in rural health ecosystems, such models promote sustainability, strengthen community engagement, and contribute to the advancement of equity in health service delivery. The success of this intervention suggests that similar educational strategies—tailored to local contexts—can serve as effective public policy tools to reduce health disparities in rural and vulnerable populations.

Replicating and adapting this model across other rural and post-conflict territories holds significant potential for informing health system reforms and fostering more inclusive, community-oriented approaches to health education and care.

## Data Availability

The datasets presented in this article are not readily available because the interviews were approved with informed consent, provided that participants’ identities remained anonymized. The same condition applied to participation in the focus group. Requests to access the datasets should be directed to jclomboc@ut.edu.co.
